# Fluoxetine treatment supports predictive validity of the three hit model of depression in male PACAP heterozygous mice and underpins the impact of early life adversity on therapeutic efficacy

**DOI:** 10.3389/fendo.2022.995900

**Published:** 2022-09-23

**Authors:** Tamás Gaszner, József Farkas, Dániel Kun, Balázs Ujvári, Gergely Berta, Valér Csernus, Nóra Füredi, László Ákos Kovács, Hitoshi Hashimoto, Dóra Reglődi, Viktória Kormos, Balázs Gaszner

**Affiliations:** ^1^ Department of Anatomy, Medical School, University of Pécs, Pécs, Hungary; ^2^ Research Group for Mood Disorders, Centre for Neuroscience & Szentágothai Research Centre, University Medical School, University of Pécs, Pécs, Hungary; ^3^ Department of Medical Biology, Medical School, University of Pécs, Pécs, Hungary; ^4^ Laboratory of Molecular Neuropharmacology, Graduate School of Pharmaceutical Sciences, Osaka University, Suita, Osaka, Japan; ^5^ Molecular Research Center for Children’s Mental Development, United Graduate School of Child Development, Osaka University, Kanazawa University, Hamamatsu University School of Medicine, Chiba University and University of Fukui, Suita, Osaka, Japan; ^6^ Division of Bioscience, Institute for Datability Science, Osaka University, Suita, Osaka, Japan; ^7^ Transdimensional Life Imaging Division, Institute for Open and Transdisciplinary Research Initiatives, Osaka University, Suita, Osaka, Japan; ^8^ Department of Molecular Pharmaceutical Sciences, Graduate School of Medicine, Osaka University, Suita, Osaka, Japan; ^9^ ELKH-PTE PACAP Research Group Department of Anatomy, Medical School, University of Pécs, Pécs, Hungary; ^10^ Department of Pharmacology and Pharmacotherapy, Medical School & Szentágothai Research Centre, Molecular Pharmacology Research Group, University of Pécs, Pécs, Hungary

**Keywords:** central amygdala, bed nucleus of stria terminalis, centrally projecting Edinger-Westphal nucleus, ventral tegmental area, dorsal raphe nucleus

## Abstract

According to the three hit concept of depression, interaction of genetic predisposition altered epigenetic programming and environmental stress factors contribute to the disease. Earlier we demonstrated the construct and face validity of our three hit concept-based mouse model. In the present work, we aimed to examine the predictive validity of our model, the third willnerian criterion. Fluoxetine treatment was applied in chronic variable mild stress (CVMS)-exposed (environmental hit) CD1 mice carrying one mutated allele of pituitary adenylate cyclase-activating polypeptide gene (genetic hit) that were previously exposed to maternal deprivation (epigenetic hit) vs. controls. Fluoxetine reduced the anxiety level in CVMS-exposed mice in marble burying test, and decreased the depression level in tail suspension test if mice were not deprived maternally. History of maternal deprivation caused fundamental functional-morphological changes in response to CVMS and fluoxetine treatment in the corticotropin-releasing hormone-producing cells of the bed nucleus of the stria terminalis and central amygdala, in tyrosine-hydroxylase content of ventral tegmental area, in urocortin 1-expressing cells of the centrally projecting Edinger-Westphal nucleus, and serotonergic cells of the dorsal raphe nucleus. The epigenetic background of alterations was approved by altered acetylation of histone H3. Our findings further support the validity of both the three hit concept and that of our animal model. Reversal of behavioral and functional-morphological anomalies by fluoxetine treatment supports the predictive validity of the model. This study highlights that early life stress does not only interact with the genetic and environmental factors, but has strong influence also on therapeutic efficacy.

## 1 Introduction

The prevalence of mood disorders like major depression rises boldly affecting more than 280 million people worldwide (1). Depression encumbers society and economy besides healthcare system, being a leading cause of chronic disability. Its early onset results in a large loss of productive life-years and a fallback in educational rate (2). Notably, suicidal attempts and completed suicide are in strong association with mood disorders (3). In spite of extended efforts to reveal the etiology and pathomechanism in the last decades, we still know only some fragments of this enormous puzzle.

An unequivocally accepted animal model would be required to inquire these processes more thoroughly. Most of the studies on animal models for depression focus on monoaminergic systems (for review see: 4–6). Nowadays, pharmacotherapy is mostly based on this concept, but increasing number of data reveal, that the therapeutic response is unsatisfactory at least in 30% of cases (7, 8). This strongly presumes that beyond the monoaminergic systems other, to date unknown mechanisms also contribute to the psychopathology. In order to obtain a new preclinical tool to clarify its background, we recently created a rodent model (9, 10) based on the widely accepted three hit theory of depression which highlights the coexistence of genetic, epigenetic and environmental factors as triggers of the disease (11, 12).

Several well-described inheritable genetic alterations (e.g. dopamine-, serotonin-, GABAergic receptor mutations) increase the probability of the disease (for review see 13–15). Increasing amount of evidence suggests that pituitary adenylate cyclase-activating polypeptide (PACAP) contributes to mood control (16, 17, for reviews see 18–22). It is known that PACAP deficiency blunts the hypothalamus-pituitary-adrenal (HPA) axis function at the level of the hypothalamic paraventricular nucleus (PVN) and adrenal cortex (23–25). Furthermore, the lack of the functional PACAP gene in knock out (KO) mice on CD1 background results in altered stress adaptation and depression-like behavior (10, 25–29). In contrast, PACAP KO mice on C57BL/6J×129SvEv (F1 hybrid) and C57Bl/6N background show an anti-depressive phenotype (30, 31). In our previous work, we found that combined stress exposure of CD1 PACAP heterozygous (HZ) mice was a suitable model for depression (10). To the best of our knowledge, no human data are available for biallelic loss-of-function mutation in the *ADCYAP1* gene linked to depression, but the recruitment of *ADCYAP1* gene polymorphisms in stress-related mood disorders (32, 33) is known. Based on this information, we decided to use the HZ mice in our three hit model, as they possess reduced PACAP content in the brain (34). The goal of this approach was to bridge the translational abyss between genetics of animal models and human population.

According to the basic concept of the three hit theory, the second ‘hit’ affects the epigenome. A well-investigated epigenetic factor is the acetylation of histone proteins (35, 36). The acetylation pattern is regulated by the balance between histone-acetyltransferase and histone-deacetylase enzyme families (37). Chronic variable mild stress (CVMS) affects the epigenome at the level of histone acetylation (38). Conversely, histone deacetylase inhibitor treatment mitigates depression-like behavior (39) while the response to antidepressant treatment is ameliorated (40, 41). These facts, on the one hand, suggest the potential contribution of epigenetic changes to the development of mood disorders by making the bed for later *noxae*, but also show that they alone do not trigger the disease. The epigenome is evolved mostly during vulnerable periods of life such as the postnatal period (42, 43). Exposure to significant adversities during this phase increase the risk of depression in animal models (44, 45) and humans (46; for review see: 47). Therefore, we (9, 10) applied maternal deprivation for modelling the effect of early life adversities on epigenome (48; for review see: 49) as the second hit.

Prolonged exposure to environmental stress plays likely the most potent role in occurrence of mood disorders (for review see: 50, 51). The normal stress adaptation response is mainly orchestrated by the HPA axis. The dysregulation of the HPA axis is well documented both in animal models for depression (10, 52–54) and in human studies (55, 56; for review see: 57). Based on these, we tested how a third hit, the chronic variable mild stress (CVMS) model of prolonged environmental challenge affects mice carrying a mutated PACAP allele (first, genetic hit) and the history of early life adversity (second, epigenetic hit) too.

As several stress-recruited brain territories contribute to the control of the HPA axis, altered top-down regulation may contribute to the pathophysiology of depression (for review see: 58). For instance, corticotropin-realasing hormone (CRH)-containing divisions of the extended amygdala (59, 60) such as central nucleus of amygdala (CeA) and oval division of the bed nucleus of the stria terminalis (ovBNST) are involved in mood control and stress response (61–65). Interestingly, they contain PACAP (66) also and manipulation by PAC1 receptor antagonist reverses the harmful effects of stress in behavioral tests (67), and their stress reactivity is compromised in PACAP KO mice (28, 68–70). The CRH-related urocortin1 (UCN1) is primarily expressed the in the centrally projecting Edinger-Westphal nucleus (cpEW) in the midbrain and these UCN1-containing cells also express PACAP mRNA (71). Their role in stress and depression models was shown in mice (10, 25, 72, 73), rats (74–77), tree shrews (62), and cpEW samples of suicide victims (73, 78) further supporting the translational value of results obtained in animal studies. Both extrahypothalamic CRH systems (79, 80) and cpEW UCN1 cells (81, 82) may interact with the serotonergic (5-HT) cells in the dorsal raphe nucleus (DR) that also show stress-induced activity (83, 84) and PACAP mRNA expression in mice (85). The high significance of serotonergic neurotransmission in mood control in the prefrontal cortex, hippocampus and amygdala is without debate (86, 87; for review see 88, 89). It is also well documented that the brain reward system and its disturbances are strongly associated with mood disorders (for review see: 90, 91). The ventral tegmental area (VTA) as the center of mesocorticolimbic dopaminergic pathway admittedly plays a crucial role in occurrence of depression (92–94).

Although the willnerian a) construct and b) face validity criteria (95) of our three hit model has already been proven (10) the primary aim of the present work was to test Willner’s third, predictive validity criterion. Therefore, we hypothesized that the treatment with a standard serotonin-reuptake inhibitor (SSRI), fluoxetine in mice carrying all three hits will reverse the depression-like state. Behavioral tests, physical and endocrinological measures, as well as functional-morphological tools in forebrain CRH-, midbrain UCN1- containing, DR-serotonergic and VTA-dopaminergic systems were used to assess the neuronal activity patterns and epigenetic alterations in the three hit model.

## 2 Methods

### 2.1 Animals and experimental design

The breeding procedure of our in-house bred PACAP KO CD1 mouse strain corresponds to the previously published protocol (10). Briefly, a HZ generation was created by crossing PACAP KO and wild type (WT) mice. Then, PACAP HZ females and males were paired on the same day. Seventeen litters born within a 72 h period were used for this study. To reduce litter differences, they were cross-fostered on postnatal day (PD) 1. Ten litters were subjected to 180 min maternal deprivation (MD180) on PD1-14: pups were separated from their dams and placed to cages lined with nesting material on a heating plate (set to 32°C) to keep them warm. Seven litters were reared according to the normal protocol of the facility (animal facility rearing, AFR). The genotype of the offspring was determined by polymerase chain reaction (PCR) using tail clipping samples collected on PD 70 (for further details on genotyping see 28).

**Figure 1:**
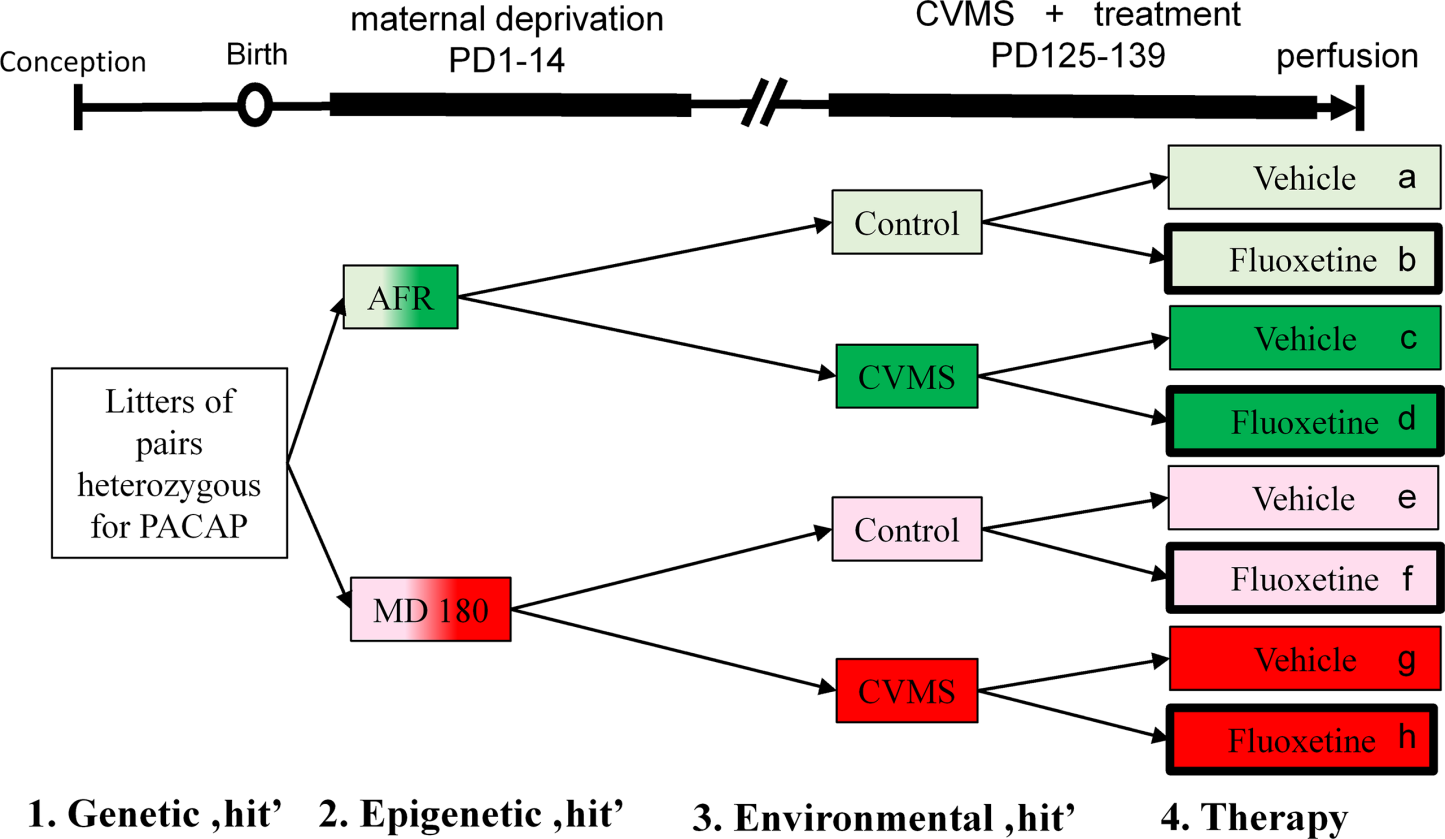
Experimental setup and timeline. Lettering (a-h) and corresponding color codes (green: animal facility-reared (AFR) groups, red: groups with history of maternal deprivation, MD180) as well as thick black frames (symbolizing fluoxetine treatment) were used to refer to the same groups throughout the text and other figure diagrams. Control (light shades) and chronic variable mild stress (CVMS)-exposed (dark shades) groups were further divided into vehicle- and fluoxetine-treated subgroups. PD, postnatal day.

Since we had previously found that in our three hit concept-based model PACAP HZ mice are ideal to use (10), we did not examine PACAP KO and WT offspring in this study. Seventeen and twenty-five male HZ mice were identified by PCR in the AFR and MD180 main groups, respectively. Four subgroups were created both in the AFR (groups were marked by a-d in the figures throughout) and MD180 subgroups (groups e-h) as shown in **Figure 1**. Four subgroups were exposed to CVMS (i.e. groups c, d and g, h) between PD125-PD139 *vs*. four control subgroups (groups a, b and e, f) that did not experience stress exposure. Half of the subgroups was treated daily by intraperitoneal (ip) fluoxetine (20 mg/kg/day in 0.2 ml saline) injections (group b, d, f, h) vs. physiological saline (0.2 ml vehicle)-injected subgroups (group a, c, e, g; see also **Figure 1**) for two weeks based on our earlier work (25). The injection site in the abdominal wall was changed daily to reduce tissue damage. The CVMS paradigm comprised mid-day (tilted cage, dark room, shaker) and overnight (wet bedding, social isolation, group holding) challenges as published previously (9, 10). Bodyweight of animals was measured on the first and on every fourth day of stress period at the time of injection procedure.

Animals were kept in standard size (30 x 30 x 28 cm) polycarbonate cages (4-5 mice per cage) on controlled temperature (24°C) and humidity (50%) with 12-hour light-dark periods (lights turned off at 6 pm) at the animal facility of Department of Anatomy, University of Pécs. *Ad libitum* access to standard rodent chaw and drinking water were provided. The wood chips litter was changed every other day.

*In vivo* experimental procedures were permitted by the National Food Chain Safety Office in Hungary (license number: BA02/2000-39/2016). The license was given based on the scientific approvals of the Animal Welfare Committee at Pécs University and the National Scientific Ethical Committee on Animal Experimentation in Hungary.

### 2.2 Behavioral tests

In total four tests were conducted in the same order in all groups as published earlier (9, 10). Light dark box (LDT) and marble burying tests (MBT) were used to assess the anxiety level. Tail suspension (TST) and forced swim tests (FST) were carried out to assess depression-like behavior. After each test, animals were placed back to their original cages. All behavioral tests were evaluated by an experienced person who was not informed about the identity of animals.

#### 2.2.1 Light-dark box test

The 40x20x27 cm box was separated into two equal compartments by a non-transparent wall with a 7x7 cm aperture. One side of the device was painted white and brightly illuminated (300 lux), while the other compartment was black-walled and dim (96). Mice were placed into the lit compartment facing to the aperture and videotaped for 5 min. We evaluated the time spent in dark compartment, number of transitions between compartments and the number of aborted transitions.

#### 2.2.2 Marble burying test

Animals were placed individually into cages (30x30x28 cm) with 24 colored marbles scattered on the surface of fresh woodchips bedding. Mice were allowed to explore the marbles for 30 min in a cage with 40 lux light intensity. The number of hidden marbles (i.e. embedded to their 2/3 into the nesting material) was registered to evaluate the anxiety level of animals (10, 97).

#### 2.2.3 Tail suspension test

Mice were suspended for 6 minutes on their tails 50 cm above a table by adhesive tape in a light room (100 lux). Cumulative time spent immobile was registered in the last 4 minutes of the video recordings (10, 98).

#### 2.2.4 Forced swim test

Mice were placed into glass cylinders (diameter: 11.5 cm; height: 25 cm) filled by 23°C tap water till the level of 19 cm based on the original description of Porsolt et al. (99) later modified by Ghasemi et al. (100). Total immobility time was evaluated in the last 4 min period of a 6 min swimming (10) test, recorded in a light room (100 lux).

### 2.3 Perfusion and sample preparation

On PD140 between 9 am and 12 pm, all animals were euthanized by urethane injection (ip; 2.4 mg/kg). All animals of a cage were injected within 2 minutes. Unconscious mice were weighed and after opening the thorax, 1 ml left ventricular blood samples were collected into syringes previously filled with 50µl 7% (w/w) EDTA solution to prevent clotting. Then, *via* the opened left ventricle, a cannula was introduced into the aorta to perfuse the animals with 20 ml of ice-cold 0.1 M phosphate-buffered saline (PBS, pH 7.4) followed by 150 ml 4% paraformaldehyde solution in Millonig buffer (pH 7.4). The right atrium was opened to allow the passage of fluids through the systemic circulation.

Blood samples were centrifuged for 5 min at 3000 rpm, and plasma supernatants were collected and stored at -20°C for corticosterone (CORT) radioimmunoassay. After perfusion, thymus and suprarenal glands were removed and weighed. Brains were dissected and postfixed at 4°C for 72 hours. Coronal sectioning was performed using a vibratome (Leica VT1000 S, Leica Biosystems, Wetzlar, Germany). Four series of 30µm sections were collected and stored in anti-freeze solution (20% ethylene glycol, 30% glycerol and 0.1 M sodium phosphate buffer) at -20°C until labeling.

### 2.4 Histology

#### 2.4.1 Free floating double-label immunofluorescence for CRH-FOSB (ovBNST and CeA), UCN1-FOSB (in cpEW), 5-HT-FOSB (in DR) and TH-FOSB (in VTA)

The FOSB protein is a product of the Fosb gene, member of the Finkel-Biskis-Jinkins murine sarcoma virus-related cellular oncogene family and it is a commonly applied marker of chronic neuronal activity (101). Sections of the brain regions to be studied were manually selected based on the mouse brain atlas by Paxinos and Franklin (102). Sections containing the ovBNST (between Bregma [Br] +0.35mm to -0.15mm) CeA (Br -1.45mm to -1.95mm), VTA (Br -2.15mm to -2.65mm), cpEW (Br -3.2mm to -3.8mm) and DR (Br -4.35mm to -4.85mm) were selected.

After removal of antifreeze solution by 6 x 10 min PBS washes, heat-induced antigen retrieval in citrate buffer solution (90°C, pH 6, 10 min) was applied. Then, 60 min 0.5% Triton X-100 (Sigma-Aldrich, St. Louis MO, USA) treatment permeabilized cell membranes. Non-specific binding sites were blocked for 60 min by normal donkey serum (NDS, Jackson Immunoresearch, Europe Ltd., Suffolk, UK) diluted in PBS to 5% for CRH-FOSB labeling and to 2% for UCN1-FOSB, 5-HT-FOSB and TH-FOSB staining procedures. Subsequently, sections were moved into the cocktail of primary antibodies solved in NDS: rabbit anti-FOSB diluted to 1:250 (Santa Cruz, sc-48, RRID:AB_631515, Santa Cruz Biotechnology Inc., Santa Cruz CA, USA) combined with a) goat anti-CRH diluted to 1:150 (Santa Cruz, sc-1759; RRID: AB_631300) for 72 h at 4°C, b) goat anti-UCN1 diluted to 1:175 (Santa Cruz, sc-1825; RRID: AB_2304014) for 48 h at 4°C, c) mouse monoclonal anti-5-HT in 1:10.000 dilution (gift from Dr. Lucienne Léger, Université Claude Bernard, Lyon, France; RRID:AB_2313872) for 48 h at 4°C, or monoclonal mouse anti-TH (1:1000, Sigma-Aldrich; RRID:AB_477569) for 16 h at 4°C. After 2 x 15 min PBS washes, sections were placed into the mixture of secondary antibodies also solved in PBS with NDS. Cyanine 3 (Cy3)-conjugated donkey anti-goat serum (1:800, Jackson Immunoresearch; RRID:AB_2340411) was used to label FOSB in all cases. To this, in order to visualize CRH immunoreaction, a biotinylated donkey anti-goat antiserum (1:1000, Jackson Immunoresearch; RRID:AB_2340397) for 24 h at 4°C was added. Following 2 x 15 min PBS washes sections were transferred into a solution of Cyanine 5 (Cy5)-conjugated streptavidin (1:1000, Jackson Immunoresearch; RRID:AB_2337245) in PBS for 3 hours. For the UCN1 labeling, Alexa 488-conjugated donkey anti-goat (1:200, Jackson Immunoresearch; RRID:AB_2336933), for 5-HT and TH, Alexa 488-conjugated donkey anti-mouse (1:500, Jackson Immunoresearch; RRID:AB_2341099) secondary antisera were added for 3 hours. Finally, sections were rinsed in PBS for 2 x 15 min, mounted on gelatin-covered slides, air-dried and ultimately covered with 50% glycerol dissolved in PBS.

#### 2.4.2 Free floating immunocytochemistry for acetyl-lysine H3 histone protein by diamino-benzidine

After 4x15 min PBS washes to remove fixative and anti-freeze solution, sections were permeabilized for 60 min in 0.5% Triton X-100 solution. Normal goat serum (NGS, Jackson Immunoresearch, 2%, in PBS) was used to block non-specific binding sites. Then, sections were moved into a solution of anti-acetyl-lysine 9 H3 histone antibodies (1:4000, Sigma-Aldrich; Cat# SAB4500347; RRID:AB_10742909) and incubated overnight at room temperature. After 2 x 15 min PBS rinses, sections were treated with biotinylated goat anti-rabbit IgG solution for 60 min (1:200, Vectastain ABC Elite Kit, Vector Lbs., Burlingame, CA, USA) followed by PBS rinses and incubation in peroxidase-conjugated avidin-biotin complex (Vectastain ABC Elite Kit) for 60 min. After further PBS washes, the immunoreaction was developed in Tris buffer (pH 7.4) with 0.02% 3,3’diaminobenzidine (DAB, Sigma-Aldrich) and 0.0003% (w/v) H_2_O_2_. To optimize the signal/background ratio, the reaction was performed under visual control using a light microscope. The reaction was stopped in PBS buffer. After PBS rinses, sections were mounted on gelatin-covered slides. After air-drying and dehydration (50%, 70%, 96%, absolute ethanol, 5 min respectively), slides were moved into xylene for 2 x 10 min and covered by Depex (Fluka, Heidelberg, Germany).

#### 2.4.3 Immunohistochemistry controls

Our CRH antibody (Santa Cruz, sc-1759) was raised against a C-terminus peptide fragment of human CRH. Based on the website of manufacturer (http://datasheets.scbt.com/sc-1759.pdf) specificity of the antibody was verified by Western blot. The UCN1 antibody (Santa Cruz, sc-1825) was raised against a C-terminus peptide fragment of rat UCN1, and we tested the specificity in our earlier works (10, 25). Our 5-HT antibody was generously provided by Dr. Lucienne Léger (Léger et al., 2001). This antibody was also tested in mouse brain tissue (25). The TH antibody (Sigma-Aldrich) was raised against C-terminus peptide fragment of mouse TH, its specificity was tested by Western blot based on the website of manufacturer (https://www.sigmaaldrich.com/catalog/product/sigma/t2928). The acetyl-lysine 9 H3 histone antibody was generated against a synthetic peptide (range of residues 3-52) containing the Lys9 acetylation site. The specificity of the serum was tested by the manufacturer in mouse tissues (https://www.sigmaaldrich.com/catalog/product/sigma/sab4500347?lang=hu&region=HU). The FOSB antibody (Santa Cruz, sc-48) used in this experiment was raised against a C-terminus peptide fragment of human FOSB also characterized earlier (10, 25, 38). The omission of either primary or secondary antisera or their replacement by non-immune sera resulted in no detectable labeling. The labeling was also prevented by preabsorption with the synthetic blocking peptides (10, 25).

### 2.5 Microscopy, digital imaging and morphometry

Study and digitalization of immunofluorescence were performed by the Olympus Fluoview 1000 confocal microscope (FV10-1000S-IX81). Images were captured by sequential scanning in photon count mode for the respective fluorophores to avoid false positive signal caused by the partial overlap of emission spectra and for detection of reliably semi-quantitative fluorescent signal. Confocal aperture settings were 80 µm, 1024 x 1024 resolution completed with a 20x objective (NA: 0.75). Excitation and emission spectra of the fluorophores were selected by applying the built-in settings of Fluoview software. To excite the dyes, the following laser beam wavelength were used: for Alexa Fluor 488, 488 nm; for Cy3, 550 nm; for Cy5, 670 nm. After scanning, pictures of channels were saved and stored both individually and superimposed using virtual red and green colors for evaluation of co-localizing fluorescent signals.

Cell counting was performed manually on non-edited images by an experienced observer who was blinded to the identity of files. Cell counts were averaged from five digital images per brain areas recorded bilaterally in the ovBNST, CeA and VTA, observing the entire territory of the respective nuclei. Considering the anatomical localization of the cpEW and DR, the evaluations were performed in the entire cross section areas next to the midline.

Intensity of immunofluorescence was measured by ImageJ software (v1.42, NIH, Bethesda, MD) evaluating 10 perikarya for CRH, UCN1, 5-HT or TH in 5 non-edited sections by manually selecting cytoplasmic areas in captures of the corresponding channel. The cytoplasmic signal density was corrected for the background signal. The latter was quantified in immunonegative territories selected randomly close to the immunoreactive cells. The calculated specific signal density (SSD) was expressed in arbitrary units (a.u.).

DAB-labeled acetyl-lysine H3 immunohistochemistry was evaluated and digitalized by a Nikon Microphot FXA microscope using a Spot RT camera (Nikon, Tokyo, Japan). Five sections of each brain area per mouse were captured. The count of marked nuclei was evaluated by manual cell counting considering the whole cross section surface area of ovBNST, CeA, cpEW, DR and VTA.

For publication purposes, selected representative images were contrasted by Photoshop software (Adobe, San Jose, CA).

### 2.6 Statistics

Statistical analysis was performed by Statistica software (v8.0; Statsoft, Tulsa, OK, USA). All data were expressed as mean and as standard error of the mean. Data beyond the two sigma range were excluded from the assessment. Normality of data was tested by Shapiro-Wilk test (103) while homogeneity of variance was evaluated by Bartlett’s Chi-square test (104). Data were subjugated to multifactorial analysis of variance (MANOVA) followed by Tukey’s *post hoc* tests (α<5%). To reveal deeper connection of datasets Spearman’s rank correlation test was performed.

## 3 Results

### 3.1 Validity of our model

#### 3.1.1 Bodyweight change

**Figure 2:**
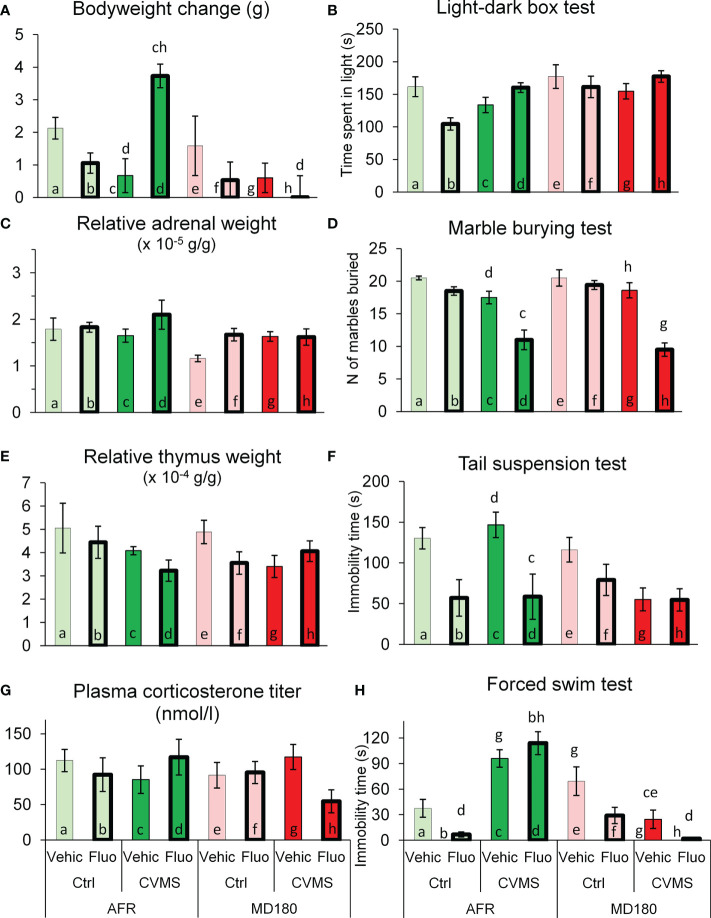
Summary of physical, endocrinological parameters and behavioral test results. **(A)** Bodyweight change of groups in the period of chronic variable mild stress (CVMS) between PD125 and perfusion (PD140), expressed in grams (g). **(C)** Relative adrenal tissue weight expressed in grams per bodyweight grams (x 10^-5^ g/g). **(E)** Relative thymus tissue weight corrected for bodyweight, expressed in grams per bodyweight grams (x 10^-4^ g/g). **(G)** Blood corticosterone titers (nmol/l). **(B)** Time spent in the illuminated compartment during light-dark box test expressed in seconds (s). **(D)** Number of marbles buried in the marble burying test. **(F)** Immobility time in tail suspension and **(H)** forced swim test, expressed in seconds (s). AFR: animal facility-reared, MD180: 180-min of maternal deprivation. Ctrl: control (i.e. not exposed to CVMS); Vehic: physiological saline; Fluo: fluoxetine. Lettering at the top of bars represents the most relevant significant *post hoc* statistical differences between pairs of groups (p<0.05).

The animals’ bodyweight change was used as an indicator of stress efficacy. MANOVA revealed the main effect of maternal care on bodyweight change in the second week of CVMS significant (F1,32 = 7.61; p<0.01). Additionally, significant second order effects as maternal care x treatment (F1,32 = 4.27; p<0.05); stress x treatment (F1,32 = 6.8; p<0.02) and a third order interaction (F1,32 = 4.36; p<0.05) of maternal care, treatment and stress were recorded. Based on *post hoc* tests, CVMS exposure resulted in blunted bodyweight gain that was reversed by fluoxetine treatment in AFR mice (see **Figure 2A**, bar c vs. d; p<0.05). In contrast, in CVMS mice with MD180 history, SSRI treatment remained ineffective on the course of bodyweight gain (**Figure 2A**, bar g vs. h). The difference between the bodyweight gain of stressed and treated animals with and without maternal deprivation is enormous (**Figure 2A**, bar d vs. h; p<0.01).

#### 3.1.2 Relative adrenal weight

MANOVA found the main effect of maternal deprivation *per se* (F1,32 = 9.38; p<0.01) and the third order interaction of factors (F1,32 = 4.57; p<0.05) on the adrenal weight significant. In contrast, the *post hoc* test did not detect significant differences between any pairs of groups (**Figure 2C**).

#### 3.1.3 Relative thymus weight

MANOVA revealed the main effect of stress (F1,34 = 4.74; p<0.04) and the triple interaction of factors (F1,34 = 11.25; p<0.01) on thymus weight significant. *Post hoc* tests showed that stress without a significant statistical power (p=0.08) decreased thymus weight in AFR fluoxetine-treated mice compared to controls (**Figure 2E**, bar b vs. d). Spearman’s rank correlation test supported the reliability of our CVMS paradigm highlighting the link between body and thymus weight data (ρ=-0.36; p<0.03).

#### 3.1.4 Corticosterone titer

The CORT titer was determined to assess the HPA-axis activity. ANOVA indicated a triple interaction of factors (F1,33 = 4.97; p<0.04). Nevertheless, stressed and fluoxetine-treated animals with MD180 history showed a tendentiously reduced CORT level, compared to vehicle-treated mice (see **Figure 2G**, bar h vs. g; p=0.15).

### 3.2 Behavioral results

#### 3.2.1 Light-dark box test

ANOVA revealed the effect of maternal deprivation (F1,33 = 7.36; p<0.02) *per se* and the interaction of stress x treatment (F1,33 = 9.0; p<0.01) significant on time spent in light compartment of the box. However, the *post hoc* tests did not identify significant differences, fluoxetine-treated control, non-deprived animals spent slightly less time in the lit compartment than vehicle-administered mice (see **Figure 2B**, bar a vs. b). In stress, this tendency was reversed (**Figure 2B**, bar b vs. d). No differences occurred across groups with the history of maternal deprivation (**Figure 2B**, see bars e-h).

#### 3.2.2 Marble burying test

Effects of stress (F1,32 = 52.0; p<10^-6^), treatment (F1,32 = 36.4; p<10^-5^) and the second order interaction of stress and treatment (F1,32 = 16.4; p<0.001) were indicated by the ANOVA. *Post hoc* tests showed decreased number of hidden marbles in case of stressed and fluoxetine-treated animals compared to vehicle-injected mice regardless the quality of maternal care (**Figure 2D**, bar c vs. d; p<0.001 and bar g vs. h; p<10^-6^).

#### 3.2.3 Tail suspension test

MANOVA found the main effect of treatment (F1,34 = 14.63; p<0.001) and the second order effects of maternal care x stress (F1,34 = 6.1; p<0.02) as well as maternal care x treatment (F1,34 = 5.62; p<0.03) significant. In mice from litters of normal maternal care, fluoxetine treatment reduced immobility time in stressed group (**Figure 2F**, bar c vs. d; p<0.05), while in controls, the effect of fluoxetine remained a tendency (bar a vs. b). In contrast, in maternally deprived offspring, the effect of fluoxetine treatment was lost, according to the *post hoc* tests.

#### 3.2.4 Forced swim test

The quality of maternal care (F1,33 = 17.6; p<0.001), the antidepressant treatment (F1,33 = 13.2; p<0.001) and the interaction of maternal care and stress (F1,33 = 64.4; p<10^-6^) affected the immobility time. *Post hoc* tests revealed that CVMS exposure tendentiously increased the immobility time in AFR mice (**Figure 2H**, bar a vs. c; p=0.11). Fluoxetine treatment was surprisingly associated also with higher immobility time (**Figure 2H**, bar b vs. d; p<0.05). Maternal deprivation changed not only the effect of stress (compare pairs of bars a and c vs. e and g), but also the efficacy of fluoxetine treatment in stressed animals: in AFR mice, the SSRI increased, while in MD180 mice it decreased the immobility time (**Figure 2H**, bar d vs. h; p<10^-4^). Fluoxetine treatment tendentiously further reduced the already low immobility time in maternally deprived stressed animals (**Figure 2H**, bar g vs. h; p=0.10).

### 3.3 Morphological results

#### 3.3.1 ovBNST

##### 3.3.1.1 Count of CRH positive neurons

**Figure 3:**
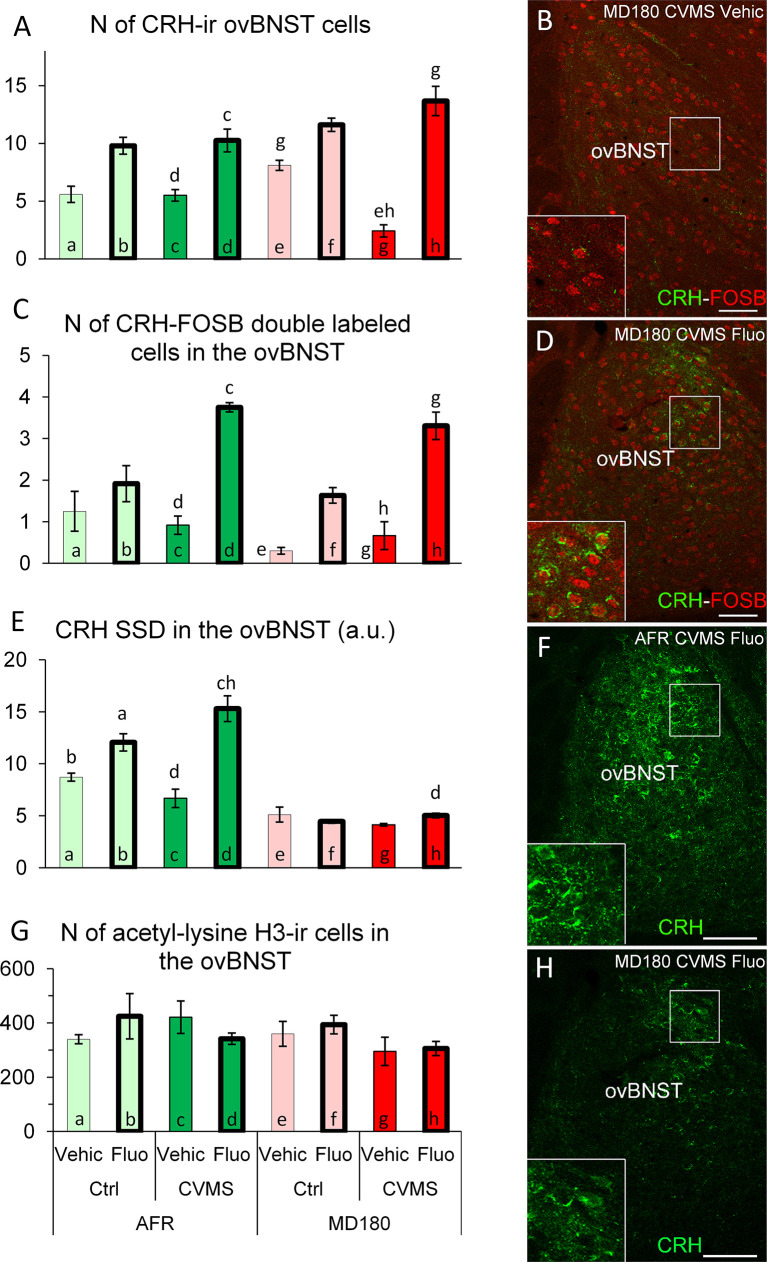
Summary of morphological results in the oval division of the bed nucleus of the stria terminalis (ovBNST). **(A)** Number (N) of corticotropin-releasing hormone (CRH)-immunoreactive (ir) cells in the ovBNST. **(C)** N of CRH/FOSB double labeled cells in ovBNST. Microphotographs **(B)** and **(D)** show CRH (green) and FOSB (red) double labeling immunofluorescence in the ovBNST. Representative images of a maternally deprived (MD180), chronic variable mild stress-exposed (CVMS), physiological saline (Vehic)-injected **(B)** and a MD180, CVMS, fluoxetine-injected (Fluo) animal **(D)**. **(E)** Specific signal density (SSD) of CRH in the ovBNST, expressed in arbitrary units (a. u.). Microphotographs of CRH (green) labeling in the ovBNST illustrate **(F)** animal facility-reared (AFR), CVMS, Fluo and an MD180, CVMS, Fluo **(H)** animals. **(G)** N of acetyl-lysine histone H3-ir cells in the ovBNST. Lettering at the top of bars represents the most relevant significant statistical differences between pairs of groups according to the *post hoc* tests (p<0.05). Ctrl: control (i.e. not exposed to CVMS). Bar = 100 µm.

Based on MANOVA, fluoxetine treatment (F1,27 = 84.3; p<10^-9^) *per se*, and the interactions of maternal care x treatment (F1,27 = 5.0; p<0.04), stress x treatment (F1,27 = 10.3; p<0.01) as well as a third order effect of maternal care x stress x treatment (F1,27 = 7.8; p<0.01) affected the CRH neuron count in ovBNST. *Post hoc* tests revealed that SSRI treatment increased the number of CRH neurons in AFR control tendentiously (see **Figure 3A**, bar a vs. b; p=0.06) and significantly in stressed animals (**Figure 3A**, bar c vs. d; p<0.05) in line with a tendency in MD180 control (**Figure 3A**, bar e vs. f; p=0.09) and a strong effect in MD180 stressed animals (**Figure 3A**, bar g vs. h; p<10^-3^). The last increment is the largest since seven times more positive cells were observed after treatment. Importantly, if mice were previously exposed to maternal deprivation, the cell number of stressed animals was decreased compared to controls (**Figure 3A**, bar g vs. e; p<0.01) in contrast to AFR mice, where no change was observed (**Figure 3A**, bar a vs. c).

##### 3.3.1.2 Count of CRH and FOSB double positive neurons

MANOVA found significant effects of maternal care (F1,27 = 5.0; p<0.04), stress (F1,27 = 17.0; p<0.001), treatment (F1,27 = 75.5; p<10^-8^) and stress x treatment interaction (F1,27 = 16.3; p<0.001). To point out the most relevant *post hoc* differences, we saw that fluoxetine treatment increased the CRH-FOSB cell count regardless if mice experienced normal maternal care (**Figures 3B–D**, bar c vs. d; p<0.001) or suffered MD180 (**Figure 3C**, bar g vs. h; p<0.001). Besides these differences, we cannot neglect that only 20-30% of CRH positive cells showed FOSB positivity.

##### 3.3.1.3 Specific signal density of CRH

The main effect of quality of the maternal care (F1,27 = 195.0; p<10^-13^) and the fluoxetine treatment (F1,27 = 39.6; p<10^-4^) as well as the second order effect of maternal care x treatment (F1,27 = 33.6; p<10^-4^) and stress x treatment (F1,27 = 14.1; p<0.001) were significant. According to the *post hoc* test, SSRI administration increased CRH-SSD both in control (**Figure 3E**; bar a vs. b; p<0.05) and stressed (**Figure 3E**, bar c vs. d; p<10^-3^) mice. Interestingly, the history of maternal deprivation completely abolished the effect of fluoxetine treatment on CRH SSD in the ovBNST (compare **Figures 3F, H**; and bars d vs. h in **Figure 3E**; p<0.01).

##### 3.3.1.4 Count of acetyl-lysine H3 positive cells

Counting of acetyl-lysine H3 positive cells revealed that the maternal care (F1,25 = 10.4; p<0.01) affected this epigenetic marker, but *post hoc* tests did not confirm any relevant differences between pairs of groups (**Figure 3G**). Notably, CORT levels of animals showed significant correlation with acetylation of the histone protein in ovBNST (ρ=0.51; p<0.01).

#### 3.3.2 CeA

##### 3.3.2.1 Count of CRH positive neurons

**Figure 4:**
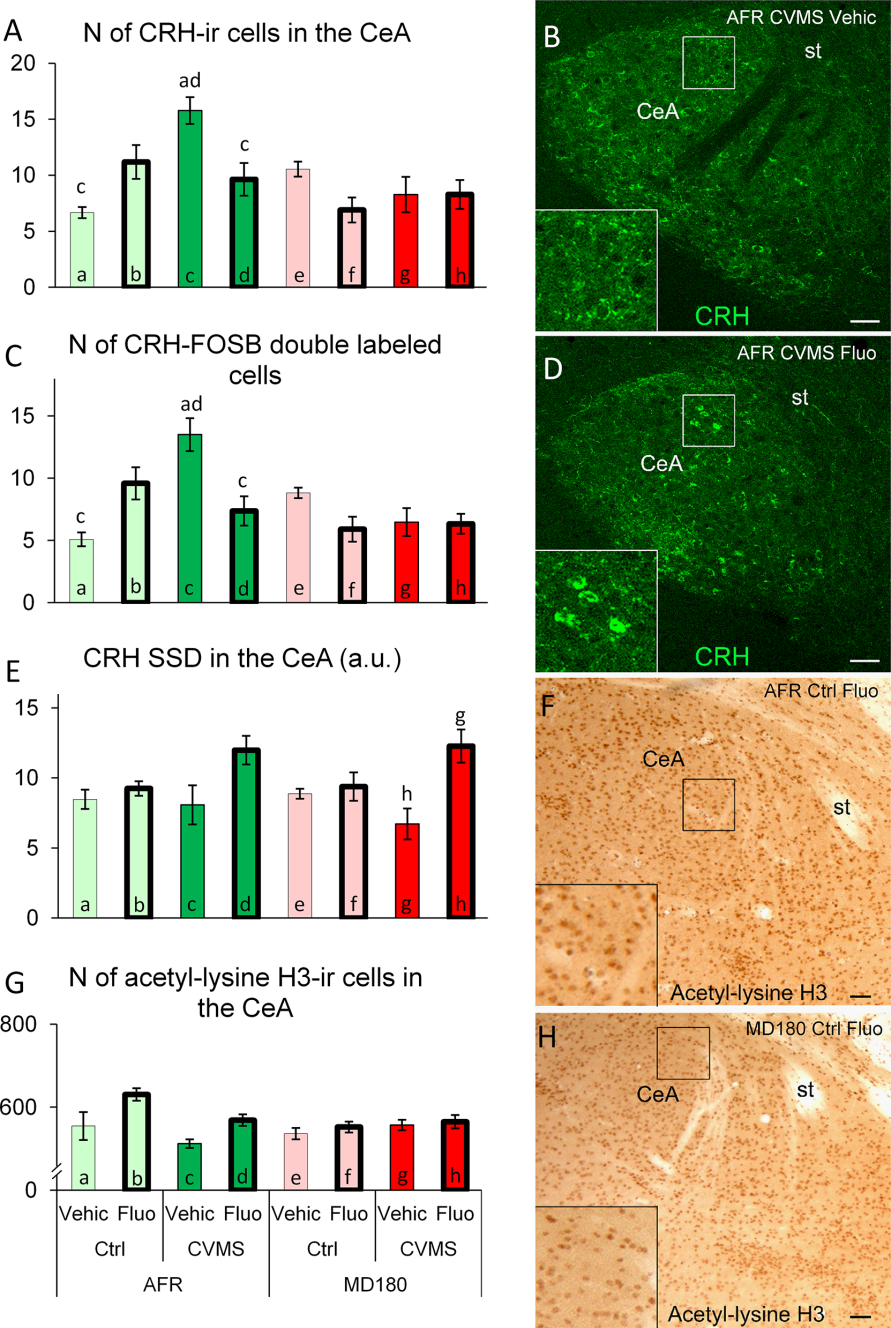
Summary of morphological results in the central nucleus of amygdala (CeA). **(A)** Number (N) of corticotropin-releasing hormone (CRH)-immunoreactive (ir) cells in the CeA. **(C)** N of CRH/FOSB double labeled cells in the CeA. Representative microphotographs of CRH (green) immunofluorescence in the CeA of an **(B)** animal facility-reared (AFR), chronic variable mild stress-exposed (CVMS), physiological saline-injected (Vehic) mouse and **(D)** an AFR, CVMS, fluoxetine injected (Fluo) animal **(D)**. **(E)** Specific signal density (SSD) of CRH in the CeA, expressed in arbitrary units (a. u.). **(G)** N of acetyl-lysine H3-ir cells in the CeA. Representative microphotographs of acetyl-lysine H3 histone immunopositive cells in the CeA of an AFR, control (Ctrl), Fluo mouse **(F)** and an MD180, Ctrl, Fluo **(H)** animal. Lettering at the top of bars represents the most relevant significant statistical differences between pairs of groups according to the *post hoc* tests (p<0.05). st: stria terminalis. Bar = 100 µm.

The main effect of maternal care (F1,25 = 7.1; p<0.02), the second order effect of maternal care x stress (F1,25 = 5.9; p<0.03) as well as the third order interaction of maternal care x stress x treatment (F1,25 = 16.9; p<0.001) were significant. CVMS exposure in AFR animals caused an approximately three-fold elevation (**Figure 4A** bar a vs. c; p<0.01; **Figures 4B, D**) in the number of CeA/CRH neurons. If these stressed animals received a fluoxetine treatment, their CRH cell count was reduced (**Figure 4A** bar c vs. d; p<0.05). A completely different pattern was observable in MD180 mice, neither stress nor CVMS exposure with fluoxetine treatment had an effect on CeA/CRH cell counts.

##### 3.3.2.2 Count of CRH and FOSB double positive neurons

MANOVA indicated the main effect of maternal care (F1,25 = 7.9; p<0.01), the second order effects of maternal care x treatment (F1,25 = 8.1; p<0.01) and stress x treatment (F1,25 = 7.6; p<0.02), as well as a third order effect of the three factors (F1,25 = 22.2; p<0.0001) on the CRH-FOSB co-localization significant. The SSRI treatment in AFR animals tendentiously elevated the count of double labeled cells in the CeA (**Figure 4C**, bar a vs. b; p=0.06) while stress exerted a statistically stronger effect (**Figure 4C**, bar a vs. c; p<0.01). However, if these stressed mice received a fluoxetine treatment, their CRH-FOSB cell count decreased (**Figure 4C**, bar c vs. d; p<0.01). Compared to AFR (**Figure 4C**, bar a and b), maternal deprivation fully abolished the effect of SSRI treatment in control mice (**Figure 4C**, bar e vs. f; p=0.33). Similarly, if the stress exposure coincided with history of maternal deprivation, the fluoxetine treatment had no effect on CRH-FOSB cell counts in the CeA.

##### 3.3.2.3 Specific signal density of CRH

Based on MANOVA, maternal care (F1,23 = 17.5; p<0.001) and interaction of stress and treatment (F1,23 = 10.1; p<0.01) had an effect on the CeA/CRH SSD. According to *post hoc* tests, in stressed animals SSRI treatment caused a tendentious increase of the cell density in AFR (**Figure 4E**, bar c vs. d; p=0.12) and a significant rise in previously maternally deprived (**Figure 4E**, bar g vs. h; p<0.01) mice.

##### 3.3.2.4 Count of acetyl-lysine H3 positive cells

MANOVA revealed the main effect of SSRI treatment (F1,26 = 9.5; p<0.01) and the second order effects of maternal care x stress (F1,26 = 7.4; p<0.02) and maternal care x treatment (F1,26 = 4.5; p<0.05) significant. SSRI treatment caused a tendency of increase in the acetyl-lysine H3 positive cell counts in AFR controls (**Figure 4G**, bar a vs. b; p=0.09) (**Figures 4F, H**) that was not detectable in animals with maternal deprivation history.

#### 3.3.3 VTA

##### 3.3.3.1 Specific signal density of TH

**Figure 5:**
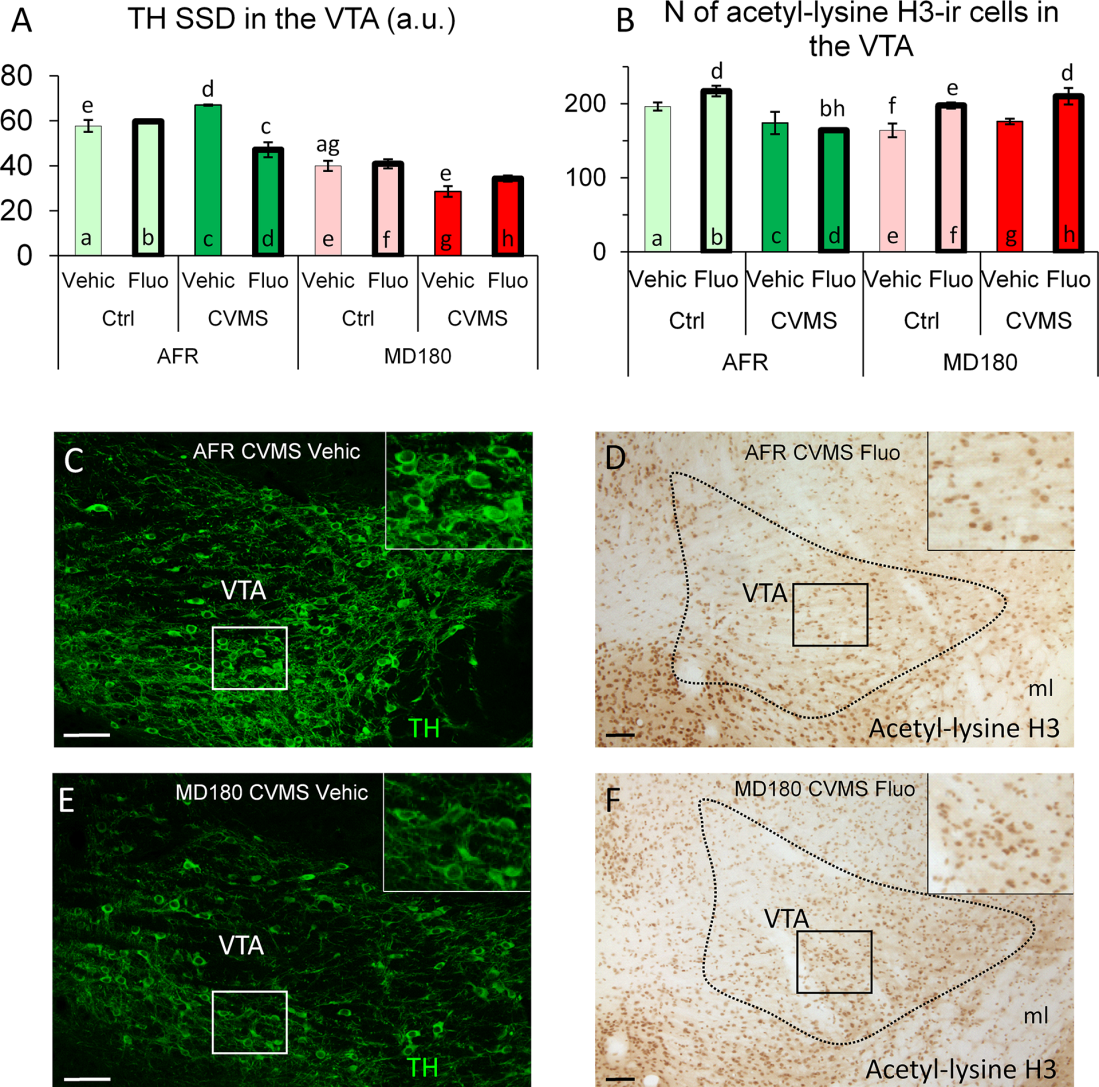
Summary of morphological results in the ventral tegmental area (VTA). **(A)** Specific signal density (SSD) of tyrosine-hydroxylase (TH) in the VTA, expressed in arbitrary units (a. u.). **(B)** N of acetyl-lysine H3 histone-immunoreactive (ir) cells in the VTA. Microphotographs of TH (green) immunofluorescence in the VTA of an animal facility-reared (AFR), chronic variable mild stress-exposed (CVMS), physiological saline-injected (Vehic) mouse **(C)** and a maternally deprived (MD180), CVMS, Vehic animal **(E)**. **(D, F)** Microphotographs of acetyl-lysine H3 histone-ir cells in the VTA of an **(D)** AFR, CVMS, Fluo and an **(F)** MD180, CVMS, Fluo animal. Lettering at the top of bars represents the most relevant significant statistical differences between pairs of groups according to the *post hoc* tests (p<0.05). Ctrl: control (i.e. not exposed to CVMS); ml: medial lemniscus. Bar = 100 µm.

MANOVA showed a significant effect of maternal care (F1,29 = 195.2; p<10^-13^) and stress (F1,29 = 11.4; p<0.01) on VTA/TH SSD. Significant second order impacts of maternal care x stress F1,29 = 5.5; p<0.03) and maternal care x treatment (F1,29 = 15.1; p<0.001) as well as a third order interaction of the three factors (F1,29 = 18.1; p<0.001) were also found. In AFR mice, fluoxetine administration reduced the TH SSD (**Figure 5A**, bar c vs. d; p<0.001) as shown by *post hoc* tests. Both control (**Figure 5A**, bar a vs. e; p<0.001) and CVMS-exposed (compare **Figures 5C, E**, moreover bar c vs. g; p<0.001 in **Figure 5E**) mice showed lower TH SSD if they previously underwent maternal deprivation which was not influenced by SSRI treatment. This lower TH SSD was further decreased by CVMS (**Figure 5A**, bar e vs. g; p<0.05). Interestingly, this is the opposite of that we observed in AFR mice (compare bar a and c vs. e and g).

##### 3.3.3.2 Count of acetyl-lysine H3 positive cells

Number of acetylated H3 histone containing cells was affected by stress (F1,26 = 4.8; p<0.04) and treatment (F1,26 = 11.5; p<0.01). Maternal care *per se* had no effect, but in interactions with stress (F1,26 = 18.4; p<0.001) and treatment (F1,26 = 6.0; p<0.03) it influenced the acetyl-lysine H3 cell count. According to the *post hoc* tests, in fluoxetine-treated AFR mice, stress reduced the cell counts (**Figure 5B**, bar b vs. d; p<0.01). Maternal deprivation tendentiously decreased the number of acetyl-lysine H3 positive cells (**Figure 5B**, bar a vs. e; p=0.11), which was reversed by SSRI administration in controls (**Figure 5B**, bar e vs. f; p=0.05), but not in stressed mice (compare **Figures 5D, F**, moreover bars d vs. h in **Figure 5E**; p<0.01). In MD180 animals following CVMS, no significant change was observed compared to controls (**Figure 5B**, bar e vs. g). The acetylation-increasing effect of fluoxetine remained below the level of significance in stressed MD180 mice (**Figure 5B**, bar g vs. h; p=0.13).

#### 3.3.4 cpEW

##### 3.3.4.1 Count of UCN1 positive neurons

**Figure 6:**
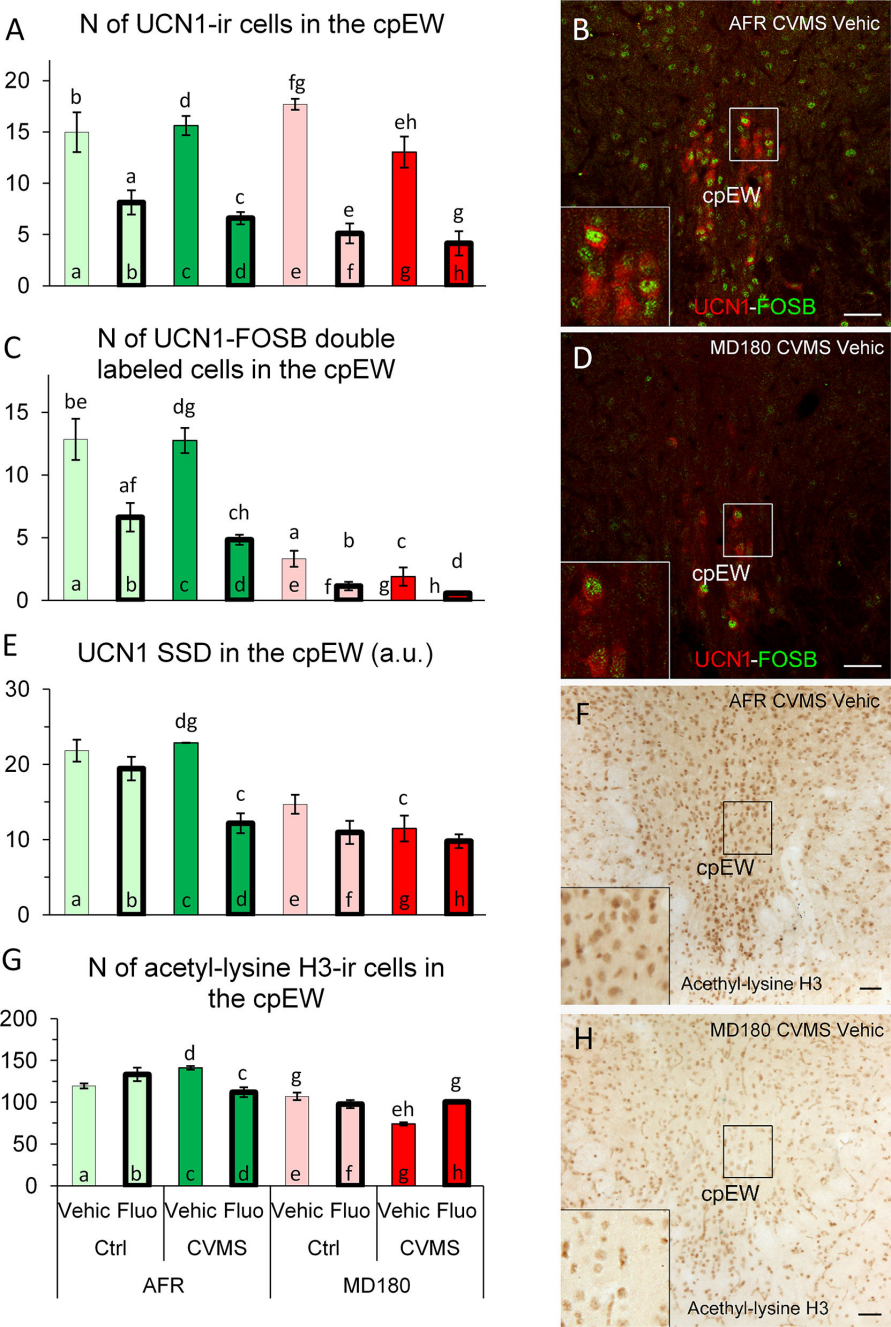
Summary of morphological results in the centrally projecting Edinger-Westphal nucleus (cpEW). **(A)** Number (N) of urocortin1 (UCN1)-immunoreactive (ir) cells in the cpEW. **(C)** N of UCN1/FOSB double labeled cells in the cpEW. **(B, D)** Representative microphotographs of UCN1 (green) and FOSB (red) double labeling in the cpEW of an **(B)** animal facility-reared (AFR), chronic variable mild stress-exposed (CVMS), physiological saline-injected (Vehic) and a **(D)** maternally deprived (MD180), CVMS, Vehic animal. **(E)** Specific signal density (SSD) of UCN1 in the cpEW, expressed in arbitrary units (a. u.). **(G)** N of acetyl-lysine H3 histone-ir cells in the cpEW. **(F, H)** Representative microphotographs of acetyl-lysine H3 histone immunopositive cells in the cpEW of an **(F)** AFR, CVMS, Vehic mouse and an **(H)** MD180, CVMS, Vehic animal. Lettering at the top of bars represents the most relevant significant statistical differences between pairs of groups, according to the *post hoc* tests (p<0.05). Ctrl: control (i.e. not exposed to CVMS); Bar = 100 µm.

The main effects of maternal care (F1,30 = 5.6; p<0.03), stress (F1,30 = 7.3; p<0.02) and treatment (F1,30 = 111.7; p<10^-10^), moreover the interaction of maternal care x stress (F1,30 = 4.7; p<0.04) and the third order interaction of the three factors (F1,30 = 6.3; p<0.02) influenced the UCN1 cell count. The fluoxetine treatment decreased the cell counts in AFR control (**Figure 6A**, bar a vs. b; p<0.01), AFR stress (**Figure 6A**, bar c vs. d; p<0.001), MD180 control (**Figure 6A**, bar e vs. f; p<0.001) and also in MD180 stress (**Figure 6A**, bar g vs. h; p<0.001) groups compared to the respective vehicle controls. Animals with maternal deprivation history also showed slightly lower cell count after stress (**Figure 6A**, bar e vs. g; p<0.05), however this difference was not visible in AFR mice (compare **Figure 6A**, bars a and c vs. e and g).

##### 3.3.4.2 Count of UCN1 and FOSB double positive neurons

The main effects of maternal care (F1,31 = 189.1; p<10^-14^), stress (F1,31 = 4.6; p<0.04) and treatment (F1,31 = 52.0; p<10^-7^), moreover the maternal care x treatment (F1,31 = 4.3; p<0.05) interaction had significant effect on UCN1-FOSB cell counts. Fluoxetine treatment reduced the UCN1-FOSB cell counts both in control (**Figure 6C**, bar a vs. b; p<0.05) and stressed (**Figure 6C**, bar c vs. d; p<0.001) AFR mice. Maternal deprivation also decreased the cell count in all comparisons with the respective AFR controls (**Figure 6C**, bars a vs. e; p<0.001, bars b vs. f; p<0.001, bars c vs. g and images **Figures 6B, D** p<0.001, moreover bars d vs. h; p<0.001).

##### 3.3.4.3 Specific signal density of UCN1

MANOVA revealed the main effects of examined factors [maternal care (F1,30 = 51.6; p<10^-7^), stress (F1,30 = 6.8; p<0.02), treatment (F1,30 = 20.5; p<10^-3^)] and their third order interaction (F1,30 = 6.4; p<0.02) significant. *Post hoc* tests showed that fluoxetine treatment in stressed AFR animals (**Figure 6E**, bar c vs. d; p<0.01) decreased UCN1 SSD. In maternal deprivation, UCN1 SSD was reduced by 50% compared to AFR (**Figure 6E**, bar c vs. g; p<0.001).

##### 3.3.4.4 Count of acetyl-lysine H3 positive cells

MANOVA revealed that the first order effects of maternal care (F1,26 = 80.8; p<10^-7^) and stress (F1,26 = 4.5; p<0.05), the second order impacts of maternal care x stress (F1,26 = 4.7; p<0.04), maternal care x treatment (F1,26 = 5.3; p<0.03), in addition, the triple interaction of factors (F1,26 = 31.1; p<10^-4^) influenced the count of acetyl-lysine H3 immunoreactive cells. According to *post hoc* tests, SSRI treatment decreased the number of acetyl-lysine H3 positive cells in AFR mice (**Figure 6G**, bar c vs. d; p<0.05). In animals with maternal deprivation history, CVMS decreased the cell count (**Figure 6G**, bar e vs. g; p<0.01) which is exactly an opposite effect compared to that observed in AFR groups (compare **Figure 6G**, bars a and c vs. e and g, as well as images **Figures 6F, H**). However, following fluoxetine treatment, cell counts returned to the level of non-stressed animals (**Figure 6G**, bar g vs. h; p<0.05).

##### 3.3.4.5 Spearman’s rank correlation

Interesting correlations were found in the functional neuromorphological variables detected in the cpEW: Acetylated histone protein cell count of cpEW showed strong positive correlations with CRH density in ovBNST (ρ=0.56; p<0.01), with UCN1-FOSB double positive cell count (ρ=0.74; p<10^-4^) and with UCN1 density (ρ=0.64; p<10^-3^) in cpEW.

#### 3.3.5 DR

##### 3.3.5.1 Count of 5-HT positive neurons

**Figure 7:**
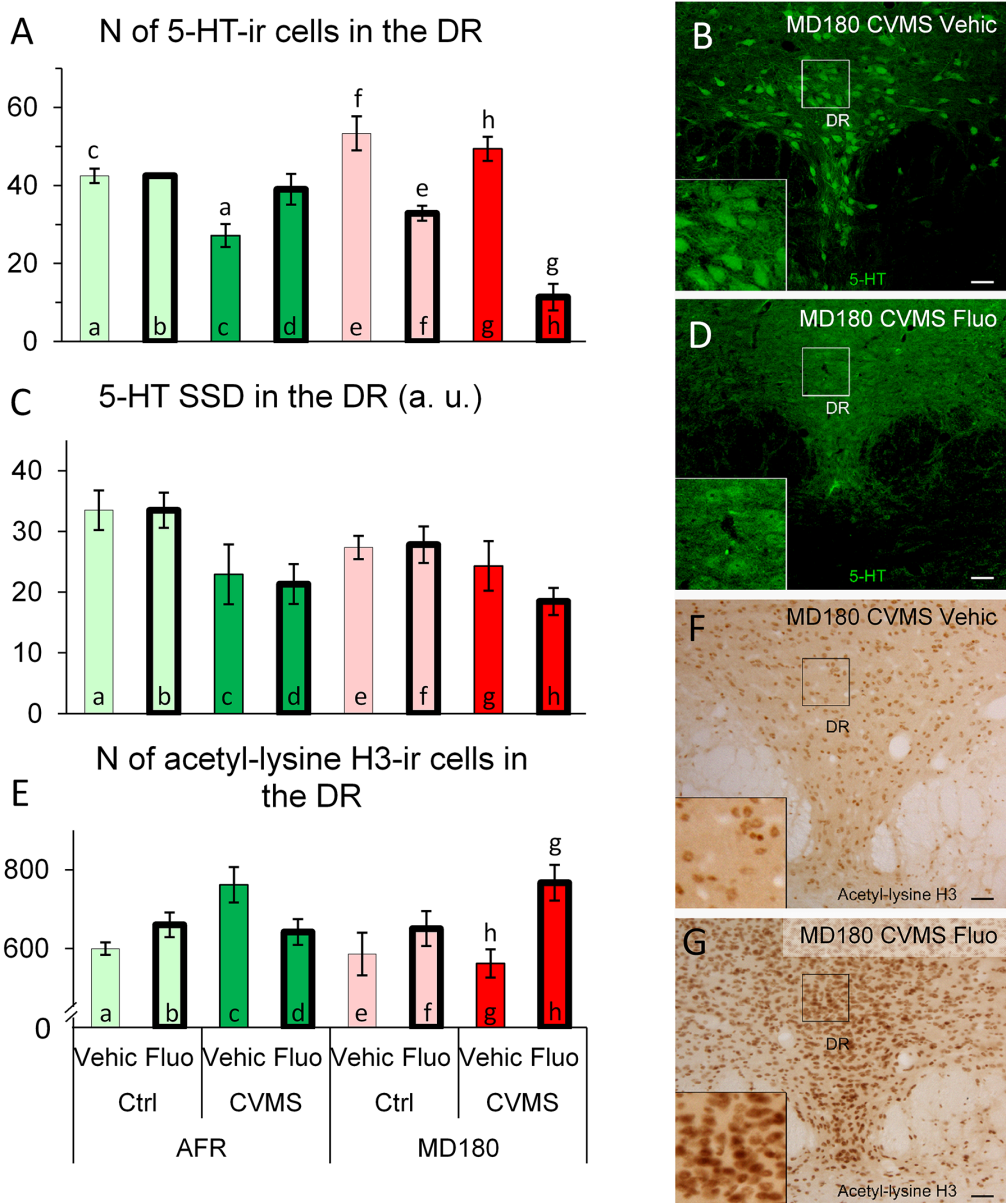
Summary of morphological results in the dorsal raphe nucleus (DR). **(A)** Number (N) of serotonin (5-HT)-immunoreactive (ir) cells in the DR. **(C)** Specific signal density (SSD) of 5-HT in the DR, expressed in arbitrary units (a. u.). Microphotographs of 5-HT (green) labeling in the DR of a **(B)** maternally deprived (MD180), chronic variable mild stress-exposed (CVMS), physiological saline-injected (Vehic) and an **(D)** MD180, CVMS, fluoxetine-injected (Fluo) animal. **(E)** N of acetyl-lysine H3 histone-ir cells in the DR. **(F, G)** Microphotographs of acetyl-lysine H3 histone positive cells in the DR of **(F)** an MD180, CVMS, Vehic mouse and an MD180, CVMS, Fluo **(G)** animal. Lettering at the top of bars represents the most relevant significant statistical differences between pairs of groups according to the *post hoc* tests (p<0.05). Ctrl: control (i.e. not exposed to CVMS); Bar = 100 µm.

MANOVA detected a significant main effect of stress (F1,25 = 26.1; p<10^-3^) and treatment (F1,25 = 29.0; p<10^-3^), moreover maternal care x treatment (F1,25 = 66.4; p<10^-7^) and a third order interaction of the three factors (F1,25 = 11.5; p<0.01) also impacted the count of DR/5-HT neurons. *Post hoc* tests revealed that among AFR animals, stress reduced the cell count (see **Figure 7A**, bar a vs. c; p<0.05) (**Figures 7B, D**) which difference disappeared if SSRI was administered (**Figure 7A**, bar a vs. d; p=0.99). Fluoxetine treatment reduced the number of 5-HT neurons in MD180 mice (**Figure 7A**, bar e vs. f; p<0.01). Stress had no further effect in MD180 mice, however treatment strongly decreased the number of 5-HT positive neurons in stressed animals with maternal deprivation history (**Figure 7A**, bar g vs. h; p<0.001). Maternal deprivation, therefore, reversed the effect of SSRI treatment in stressed animals (compare **Figure 7A**, bars c and d vs. g and h).

##### 3.3.5.2 Count of 5-HT and FOSB double positive neurons

Examining the cell count by MANOVA neither FOSB, nor 5-HT and FOSB double positive neurons showed significant effect at any of the factors or their interactions.

##### 3.3.5.3 Specific signal density of 5-HT

MANOVA indicated the main effect of stress (F1,25 = 11.9; p<0.01) on 5-HT SSD in the DR. *Post hoc* tests did not identify any significant differences between pairs of groups (Figure 7C).

##### 3.3.5.4 Count of acetyl-lysine H3 positive cells

MANOVA revealed a significant effect of maternal care x treatment (F1,28 = 6.6; p<0.02) and the third order effect of maternal care x stress x treatment (F1,28 = 6.3; p<0.02) interaction on acetyl-lysine H3 immunoreactivity significant. *Post hoc* tests represented that count of acetylated H3 histone-containing cells was higher in stressed MD180 mice following SSRI treatment (**Figure 7E**, bar g vs. h p<0.05; **Figures 7F, G**).

##### 3.3.5.5 Spearman’s rank correlation

Acetylation of histone H3 in the DR correlated positively with CRH positive (ρ=0.47; p<0.01) and CRH-FOSB double positive cell count (ρ=0.46; p<0.01) in ovBNST. While the correlation was negative with the number of UCN1 positive neurons in cpEW (ρ=-0.46; p<0.01) and the count of 5-HT positive cells of DR (ρ=-0.40; p<0.04).

### 3.4 Correlation between behavioral and morphological observations

Time spent in the lit compartment during LDT showed negative correlation with histone acetylation of CeA (ρ=-0.40; p<0.02). MBT-anxiety level correlated with cpEW/UCN1 (ρ=0.36; p<0.03), 5-HT/DR (ρ=0.41; p<0.02) cell counts, and with 5-HT/DR SSD (ρ=0.59; p<0.001). Moreover, a negative correlation of anxiety level with ovBNST CRH/-FOSB cell count (ρ=-0.54; p<0.001) and with CRH/CeA SSD (ρ=-0.58; p<0.001) was detected.

TST immobility time correlated negatively with the CRH/ovBNST cell count (ρ=-0.46; p<0.01) and positively with the UCN1/cpEW cell number (ρ=0.46; p<0.01). FST immobility time showed a strong negative correlation with histone acetylation of VTA (ρ=-0.57; p<0.001) and a similarly strong, but positive connection with the bodyweight change (ρ=0.54; p<0.001).

## 4 Discussion

### 4.1 Validity of our model

After testing the construct and face validity (10) of our animal model for the three hit theory of depression, here we aimed at investigating the third predictive validity criterion by Willner (95). Compared to our previous studies (9, 10) we supplemented the experimental setup with a cohort of fluoxetine-treated PACAP HZ mice to test if the animals showing depression-like phenotype and carrying all three ‘hits’ respond to the SSRI treatment. With this strategy, we have successfully reproduced the results supporting the construct and face validity criteria by assessment of physical parameters and behavioral tests and we have also verified (**Table 1**) the predictive validity of our model.

**Table 1:** Summary of maternal deprivation effects on stress and treatment.

			AFR		MD180	
			Stress	Treatment	Stress	Treatment
HPA axis response	Physical parameters	Bodyweight gain	●	●	●	●
		Adrenal weight	●	●	●	●
		Thymus weight	●	●	●	●
	Endocrine parameter	CORT	●	●	●	↓
Behavioral tests	anxiety	MBT	●	↓	●	↓
		LDT	●	●	●	●
	depression level	FST	↑	●	↓	↓
		TST	●	↓	↓	●
Morphology	ovBNST	CRH+	●	↑	↓	↑
		Coloc with FOSB	●	●	●	↑
		CRH SSD	●	↑	●	●
		Histone	●	●	●	●
	CeA	CRH+	↑	↑	●	●
		Coloc with FOSB	↑	↑	●	●
		CRH SSD	●	●	●	●
		Histone	●	↑	●	●
	VTA	TH SSD	↑	●	●	●
		Histone	●	●	↑	●
	cpEW	UCN1+	●	↓	↓	↓
		Coloc with FOSB	●	↓	●	↓
		UCN1 SSD	●	●	●	●
		Histone	↑	●	↓	●
	DR	5-HT	↓	●	●	↓
		5-HT SSD	↓	●	●	●
		Histone	●	●	●	●

Yellow: effect of stress OR treatment was affected by maternal deprivation. Red cells: maternal deprivation affected BOTH stress AND treatment effects. Dark background refers to paradoxical/maladaptive changes. ↑ increase, ↓ decrease, • no effect according to the post hoc tests. Within the main category ‘AFR’, in the ‘Stress’ column, the AFR control vehicle group was compared with the AFR stress vehicle group. In ‘Treatment’ column, the AFR control vehicle group was compared with AFR control fluoxetine animals. In the main category ‘MD180’, in the ‘Stress’ column, the MD180 control vehicle group was compared with the MD180 stress vehicle group. In ‘Treatment’ column, the MD180 control vehicle group was compared with MD180 control fluoxetine animals. AFR, animal facility-reared; MD180, maternally deprived; HPA axis, hypothalamus-pituitary-adrenal axis; ovBNST, oval division of bed nucleus of stria terminalis; CeA, central nucleus of amygdala; VTA, ventral tegmental area; cpEW, centrally projecting Edinger-Westphal nucleus; DR, dorsal raphe nucleus; CORT, corticosterone titer; MBT, marble burying test; LBD, light-dark box test; FST, forced swim test; TST, tail suspension test; CRH+, corticotropin-releasing hormone immunopositive cell count; SSD, specific signal density; Coloc with FOSB, histological co-localization of the given antigen and FOSB; TH, tyrosine-hydroxylase, UCN1+: count of urocortin1 immunoreactive cells, 5-HT+: count of serotonin immunoreactive cells, Histone: acetyl-lysine H3 histone immunoreactivity.

The efficacy of CVMS was supported by the flattened bodyweight gain curve (**Figure 2A**) in the second week of the stress period. This is in line with our data (10, 25) and others’ earlier results (105–108; for review see: 6). Epigenetic alterations were associated with decreased bodyweight gain, which is also in agreement with other animal (109) and human studies (110). This supports our successful intervention in the early life establishment of epigenetic programming in our animals. In order to prove this experimentally, we expanded our morphological work with the quantitation of an epigenetic marker, acetyl histone H3.

Relative adrenal weight and thymus data further supported the overall effectiveness of maternal deprivation as stressor as shown by ANOVA, even though the *post hoc* tests did not detect significant difference between the pairs of groups that is in line with findings in a maternal deprivation model (111). This might have required a longer stress period (112–114) but the animals’ individual stress sensitivity may have also abolished the significance in some cases (115).

However, after maternal deprivation and stress, SSRI treatment slightly decreased the CORT level, this was statistically not significant. It is known from animal (116) and human (117) data that the over-activity of the HPA axis may be normalized by antidepressants. Since the HPA axis over-activity (118, 119) is characteristic only for a subpopulation of depressed patients, an intervention into the HPA axis offers remarkable therapeutic advantage only in this group of cases (120). In this experiment, mice underwent an anesthesia before the perfusion procedure. The acute stress effect on the CORT titer might have caused a relatively large error of CORT data abolishing significant differences. On the other hand, a longer period of SSRI treatment could cave induced a larger effect, however the we found in a similar experimental setup (25) that in two weeks the anti-depressive effect is already well detectable.

### 4.2 Behavioral considerations

Results of MBT obviously support the predictive validity of our model because regardless the quality of maternal care, fluoxetine treatment effectively reduced the anxiety level of stressed animals. The anxiety-reducing effect of fluoxetine in MBT was not detected in the AFR control group. This is in line with earlier results suggesting a slight anxiogenic effect of fluoxetine administration in non-stressed mice (121). In agreement with this, fluoxetine reduced the anxiety level of stressed mice only as it was also found by other laboratories (122). A similar tendency of fluoxetine effect was observed in the LDT while the history of maternal deprivation fully abolished the efficacy of fluoxetine. This further underlines that early life adversity affects stress adaptation and response to antidepressant treatment (for review see: 123–125). In our model, TST showed higher sensitivity than FST. FST showed smaller differences between certain groups and higher depression level after fluoxetine treatment in AFR stressed mice. This second finding turns practically against all other results. Findings of other laboratories are in agreement with the higher sensitivity of TST (126), while recently, more and more established researchers are on the opinion that FST should no longer be used to measure depression level (127–130). According to the TST, SSRI treatment potently reduced depression level in AFR animals. History of maternal deprivation (without CVMS) blunts the effectivity of fluoxetine treatment. If all risk factors coincided, animals failed to adapt (10, 12). These behavioral findings ultimately underpin the significance of personalized therapy in mood disorders (131) that considers the number and type of risk factors that the patient carries.

In this study we examined the interactions between adverse early life event and stress exposure. We cannot rule out that the stress effect of the behavioral tests interacted with each other and presumably also with the MD180 and CVMS ultimately affecting our results. Nevertheless, in order to keep the conditions as standard as possible we applied the tests in the same order in all groups on consecutive days.

### 4.3 Morphological findings

#### 4.3.1 ovBNST

In contrast to our previous findings, CVMS exposure did not induce significant activation in ovBNST among AFR animals (10) which may be explained by the blunting effect of mild stress caused by daily intraperitoneal injections of vehicle animals. However, following maternal deprivation, the magnitude of response to the same stress in both CRH and FOSB cell counts decreased. This supports the conclusion of our behavioral tests, where we saw maladaptation in deprived animals. Our correlation analyses further supported that anxiety (in MBT) and depression levels (in TST) are inversely correlated with FOSB positivity in ovBNST/CRH cells, in agreement with previous findings in rats (61). In line with this, the fluoxetine treatment-induced elevation of CRH/FOSB cell counts in most of the groups. This was associated with potent anxiolytic and antidepressant effect in behavioral tests. Increased ovBNST/CRH cell count upon fluoxetine treatment is in full agreement with results of other studies at protein and mRNA level (132–135). CRH SSD measurements revealed very low ovBNST/CRH content in all deprived groups, suggesting that maternal deprivation reduces CRH content of these cells in line with the results of others (136). However, in the ovBNST these changes have no apparent epigenetic background, as revealed by acetylation of histone H3.

#### 4.3.2 CeA

The CeA, usually activated by stress (62–65) also showed elevated CRH-FOSB count, which was concomitant with higher CRH content suggesting increased activity. Fluoxetine treatment following stress decreased the number of CRH positive neurons. Similar to the ovBNST, SSRI treatment in AFR control mice tendentiously increased the number of CRH-producing neurons that is in agreement with earlier findings (137). In line with other brain areas described above, history of maternal deprivation fundamentally rearranged the neuronal activity pattern in control and stressed PACAP HZ mice, because fluoxetine treatment decreased the activity and CRH content of CeA, and stress failed to activate the nucleus or increase the count of CRH-producing cells. This phenomenon repeatedly supports, that early life adversity caused maladaptive changes at neuronal stress responsivity which ultimately contributed to the anomalies observed behavioral tests as others also proved (138). Negative correlation between CeA/CRH SSD and number of hidden marbles in MBT demonstrates both the potent anxiolytic effect of fluoxetine after stress (135), and that higher CeA/CRH level is associated with lower anxiety. This finding seems to be paradoxical as higher CRH levels are usually associated with increased anxiety (139), but if early life adversities influence the response, it is not without example that the opposite was observed (140).

#### 4.3.3 VTA

VTA plays a crucial role in activity of mesolimbic pathway impacting symptoms of mood disorders like anhedonia and reduced motivation (92). As dopamine is the key neurotransmitter in this system, we examined the immunoreactivity of TH, the rate-limiting enzyme, of dopamine synthesis. Marking tyrosine hydroxylase (TH) is a traditional tool for investigating its activation and estimate the pursuit of mesocorticolimbic pathway (141, 142). The effect of maternal deprivation was similar in this nucleus to that observed the extended amygdala. Moreover, TH SSD was decreased by 40% in offspring that suffered maternal deprivation fitting with earlier findings (93). These data further highlight that early life adversities cause long-term changes in stress adaptation and in responsivity to antidepressant therapy (124, 136; for review see: 47, 143). Although, we did not detect that H3 histone acetylation would interact with TH SSD, we cannot exclude that other, here not examined epigenetic mechanisms contributed to the long-term changes in VTA TH SSD. Consonantly with earlier results (40, 41) only fluoxetine treatment increased the acetylation. This way, fluoxetine may increase the feeling of reward thus mitigating the previously described symptoms like anhedonia and lack of motivation (92).

#### 4.3.4 cpEW

The response of UCN1-producing neurons to fluoxetine treatment was uniform in all groups: they showed decreased UCN1 content. This, in correlation with the results of MBT establish an ensuing evidence of the predictive validity of our model and supports the importance of cpEW in anxiety-related diseases in line with earlier published data (25, 28, 62, 73, 74, 76, 77, 144). Early life stress did not affect the urocortinergic cell count, but decreased their FOSB activity and UCN1 content. It is consonant with the results of acetylation of histone proteins and the correlation between the acetylation and UCN1 density, where lower acetylation was coupled with lower SSD. A further correlation between histone acetylation in the cpEW and CRH density in ovBNST highlights the role of urocortinergic system in the regulation of BNST (71) and the HPA axis (145). The observation, that in contrast to AFR animals, stress exposure in maternally deprived animals resulted in the lowest acetylation level, suggests profound maladaptive changes at neuronal level. That may contribute to the behavioral alterations following maternal deprivation and it is also in line with our earlier results (76) where effects of maternal deprivation were similar in a rat model. Importantly, this lowest acetylation level was increased by fluoxetine treatment, in line with the findings in frontal cortex (40). This fact repeatedly highlights the predictive validity of our model.

#### 4.3.5 DR

DR/5-HT neurons serve as important regulators of HPA axis and limbic brain territories in mood regulation (146). The behavioral anomalies observed in PACAP KO mice are attributed to alterations of the serotonergic system (147). We found here that in AFR mice, stress decreased both the DR/5-HT cell count and 5-HT SSD in line with our (25, 28) and other’s previous results (145). Importantly, after fluoxetine treatment the 5-HT cell count did not differ from the control (148, 149). Following maternal deprivation, an elevated cell count was shown consonantly to the results of other laboratories (150). In maternally deprived mice, stress *per se* did not affect DR/5-HT, while the effect of fluoxetine decreased the DR/5-HT cell counts. This further supports the significance of early life adversities in stress adaptation response and mood control in line with several other studies (for review see: 151–153).

The count of hidden marbles in MBT showed a negative correlation with the number of DR/5-HT cells and their 5-HT SSD. This strongly suggests a true link between our morphological and behavioral findings. This is in line with a large body of evidence underlining the role of the serotonin systems both in mood control and mood disorders (86, 87, 147, for review see: 88, 89). In mice with history of maternal deprivation, stress caused no change, but fluoxetine administration in CVMS increased histone acetylation. In line with this, higher epigenetic modification upon antidepressant administration was observed by Levine et al. (40) also. The strong negative correlation of 5-HT positive cell counts and the number of acetylated histone-containing cells of DR highlights the importance of epigenetics in mood control. The strong correlation between 5-HT cell counts and ovBNST/CRH as well and cpEW/UCN1 cell count suggests a functional connectivity, that is in agreement with earlier neuroanatomical studies by Waselus et al. (154, 155) and Priest et al. (71).

#### 4.3.6 Correlation between behavioral and functional morphological findings

In this study we performed functional neuroanatomical studies in a number of limbic centers and found that all of the examined brain areas may have contributed to the altered mood status. The ovBNST and CeA CRH activity was inversely, while the cpEW/UCN1 and DR/5-HT directly correlated with MBT anxiety and TST depression levels suggesting their opposing contribution to the control of mood status (for review see: 19). Histone acetylation in the VTA correlated with the FST behavior suggesting the long term contribution of this area to mood status.

### 4.4 Limitations

The brain areas studied here are highly estrogen sensitive (156–161). Therefore, one has to consider the behavioral and even functional-morphological effect of the actual estrus cycle phase in female animals. Collecting vaginal smear samples to monitor the cycle would be an additional stress factor and the CVMS exposure might also interact with the cycle. Taking the complexity of the animal experimental setup, the capacity of our animal facility and the above described considerations in account, we waived to test the model in female mice that is an important limitation of this study.

In this experiment we did not apply wild type and PACAP knockout animals. Therefore, in this study we did not test if the reduced amount of PACAP (26) *per se* contributes to the depression-like phenotype, however this has been shown earlier in our (9, 10) and in other laboratories (26, 27, 147) also.

### 4.5 Conclusions and future perspective

Based on the three-hit concept of human depression (11, 12) we recently developed a model for depression studies, using PACAP heterozygous mice subjected to maternal deprivation and chronic variable mild stress and proved the willnerian (95) construct and face validity criteria (9, 10). Our present results demonstrate the reproducibly of our earlier results. The third, willnerian predictive validity criterion was tested in this study successfully as fluoxetine treatment restored MBT anxiety and TST depression level.

Our morphological data suggest that maternal deprivation as the model of early life adversity applied in genetically vulnerable PACAP heterozygous mice causes fundamental brain area-specific changes in the histone acetylation and stress adaptation response of the CRH-containing nuclei of the extended amygdala, dopaminergic VTA, UCN1-expressing cpEW and the serotonergic DR.

The complexity of the observed functional-neuromorphological alterations (**Table 1**) further supports the validity of our model taking the recruitment of many neurotransmitter and neuromodulator systems in consideration [for review see: (162)]. Further systematic brain mapping studies are required to identify brain areas or neural circuits, which contribute to some specific aspects of mood disorder neurobiology. Testing of candidate antidepressant compounds in this model may help to find new therapeutic strategies for the management of stress-related mood disorders.

## Data availability statement

The raw data supporting the conclusions of this article will be made available by the authors, without undue reservation.

## Ethics statement

*In vivo* experimental procedures were permitted by the National Food Chain Safety Office in Hungary (license number: BA02/2000-39/2016). The license was given based on the scientific approvals of the Animal Welfare Committee at Pécs University and the National Scientific Ethical Committee on Animal Experimentation in Hungary.

## Author contributions

TG performed the animal experiments evaluated the results and statistics, wrote the first draft of the manuscript. DK, BU, NF, LK performed the animal experiments, immunolabelings and cell counting. JF performed and analyzed the behavioral results. VC performed CORT RIA measurements, analyzed and validated the results. GB contributed to the confocal imaging and morphometrical analyses. HH and DR provided the genetically modified mice and contributed to the manuscript. VK contributed to the planning of study design, result assessment and writing. BG created the study design, contributed to the animal experiment, tissue sampling, imaging, data analysis and statistics, prepared the figures and supervised the manuscript. All authors contributed to and approved the final version of the manuscript.

## Funding

This project (TKP2021-EGA-16) has been implemented with the support provided from the National Research, Development and Innovation Fund of Hungary, financed under the TKP2021-EGA funding scheme and also under the 2020-4.1.1-TKP2020 funding scheme (Project No: TKP2020-IKA-08). The work was also supported by the Hungarian Scientific Research Fund (NKFIH, PD100706 and FK124188) to BG. VK was sponsored by the research grant of the Medical Faculty, University of Pécs (KA-2019-12), by National Research, Development and Innovation Fund (ÚNKP-22-5-PTE-1740) and by the János Bolyai Research Scholarship of the Hungarian Academy of Sciences (BO/00750/22/5). NF was supported by the research grant of Pécs University Medical School KA-2020-03, and New National Excellence Program of the Ministry for Innovation and Technology from the source of the National Research, Development and Innovation Fund (ÚNKP-20-4-II-PTE-547). This work was also financed by NAP 2017-1.2.1-NKP-2017-00002; MTA-TKI14016.

## Acknowledgments

Authors are thankful for their excellent technical help to Beatrix Brumán and Izabella Orbán.

## Conflict of interest

The authors declare that the research was conducted in the absence of any commercial or financial relationships that could be construed as a potential conflict of interest.

## Publisher’s note

All claims expressed in this article are solely those of the authors and do not necessarily represent those of their affiliated organizations, or those of the publisher, the editors and the reviewers. Any product that may be evaluated in this article, or claim that may be made by its manufacturer, is not guaranteed or endorsed by the publisher.
